# The role of nitric oxide in the interaction of *Arabidopsis thaliana* with the biotrophic fungi, *Golovinomyces orontii* and *Erysiphe pisi*

**DOI:** 10.3389/fpls.2013.00351

**Published:** 2013-09-09

**Authors:** Markus Schlicht, Erich Kombrink

**Affiliations:** Chemical Biology Laboratory, Max Planck Institute for Plant Breeding ResearchKöln, Germany

**Keywords:** disease resistance, plant defense signaling, plant immunity, plant-microbe interaction, powdery mildew, *Golovinomyces orontii*, *Erysiphe pisi*

## Abstract

Powdery mildews are a diverse group of pathogenic fungi that can infect a large number of plant species, including many economically important crops. However, basic and applied research on these devastating diseases has been hampered by the obligate biotrophic lifestyle of the pathogens, which require living host cells for growth and reproduction, and lacking genetic and molecular tools for important host plants. The establishment of *Arabidopsis thaliana* as a host of different powdery mildew species allowed pursuing new strategies to study the molecular mechanisms governing these complex plant–pathogen interactions. Nitric oxide (NO) has emerged as an important signaling molecule in plants, which is produced upon infection and involved in activation of plant immune responses. However, the source and pathway of NO production and its precise function in the regulatory network of reactions leading to resistance is still unknown. We studied the response of *Arabidopsis* *thaliana* to infection with the adapted powdery mildew, *Golovinomyces orontii* (compatible interaction) and the non-adapted, *Erysiphe pisi* (incompatible interaction). We observed that NO accumulated rapidly and transiently at infection sites and we established a correlation between the resistance phenotype and the amount and timing of NO production. *Arabidopsis* mutants with defective immune response accumulated lower NO levels compared to wild type. Conversely, increased NO levels, generated by treatment with chemicals or expression of a NO-synthesizing enzyme, resulted in enhanced resistance, but only sustained NO production prevented excessive leaf colonization by the fungus, which was not achieved by a short NO burst although this reduced the initial penetration success. By contrast, lowered NO levels did not impair the ultimate resistance phenotype. Although our results suggest a function of NO in mediating plant immune responses, a direct impact on pathogen growth and development cannot be excluded.

## INTRODUCTION

The sessile lifestyle of plants makes it impossible for them to escape from environmental pressures. To avoid biotic stresses and colonization by microbial pathogens, such as fungi, bacteria, or viruses, plants have evolved a multitude of rapid and efficient defense mechanisms. They are guided by the ability to sense pathogen attacks and to translate this perception into an adaptive defense response. Following the detection of a pathogen *via* highly conserved microbe- or pathogen-associated molecular pattern (MAMPs or PAMPs), such as elicitor-active epitopes of bacterial flagellin (flg22) or fungal chitin, and the corresponding plasma membrane-localized pathogen pattern recognition receptors (PRR), numerous signaling molecules are released, including reactive oxygen species (ROS), calcium ions, salicylic acid (SA), jasmonic acid (JA), and nitric oxide (NO), which are thought to mediate the activation of powerful immune responses (Chisholm et al., 2006; Jones and Dangl, 2006; Boller and Felix, 2009). This PAMP-triggered immunity directed against non-adapted pathogens is also referred to basal or non-host resistance. As a mechanism to counteract plant defense mechanisms, host-adapted pathogens have acquired the capacity to escape from recognition and/or to produce effectors that suppress PRR-triggered plant defenses (Göhre and Robatzek, 2008; Deslandes and Rivas, 2012; Rafiqi et al., 2012). Plants in turn evolved a second system of immune sensors, so-called resistance (R) proteins that are localized inside plant cells and recognize pathogen effectors thereby activating an even stronger immune response (Takken and Goverse, 2012). This effector-triggered immunity shares numerous signaling and downstream components with PAMP-triggered immunity (Chisholm et al., 2006; Jones and Dangl, 2006). R protein-mediated, effector-triggered immunity typically involves defense gene activation and the hypersensitive cell death response (HR) at the site of attempted host colonization (Stuible and Kombrink, 2004; Williams and Dickman, 2008; Coll et al., 2011). The outlined dual plant defense system provides resistance against a wide variety of pathogens and only a few adapted pathogens can successfully circumvent or suppress both defense layers and cause disease.

The causal agent of the powdery mildew disease encompasses a diverse range of pathogenic fungi (order Erysiphales) that are widespread, obligate biotrophic plant pathogens colonizing a large number of different plant species, including many economically important crops (Micali et al., 2008). With the relatively recent identification of powdery mildew species that are pathogenic on *Arabidopsis thaliana*, additional tools and experimental strategies have become available to study these complex pathogens and their interaction with this model host plant. This includes structural and functional changes that occur during host colonization, mechanisms of defense signaling/initiation and identification of genetic components responsible for compatibility and incompatibility, which may help to develop successful crop protection strategies and new agricultural practices (Micali et al., 2008).

For successful host colonization, powdery mildew conidiospores germinating on the leaf surface have to breach the epidermal cell walls, which is the first critical step of the infection process and requires formation of the appressorium and infection peg. Subsequently, the plant plasma membrane invaginates and the haustorium develops, which finally forms as branched unicellular body and functions as the intracellular feeding structure (Koh et al., 2005; Micali et al., 2008). Such established fungus can form colonies and complete the life cycle by producing conidiophores and spores for new infection. Few powdery mildew species are able to infect *Arabidopsis thaliana*, including *Golovinomyces cichoracearum *and* Golovinomyces orontii*, which are pathogens of cucurbits and crucifers (Plotnikova et al., 1998; Saenz and Taylor, 1999; Vogel and Somerville, 2000). By contrast, *Arabidopsis* is resistant to non-adapted powdery mildews, such as *Blumeria graminis *f. sp. *hordei* (pathogenic on barley) or *Erysiphe pisi* (pathogenic on pea), and this non-host resistance is readily detectable at the penetration stage by arrest of most host cell entry attempts (usually >80%). Rare cases of haustorium formation are usually accompanied by timely callose encasement and the HR of attacked epidermal cells, which prevents further fungal development (Collins et al., 2003; Lipka et al., 2005; Stein et al., 2006; Hardham et al., 2007). Thus, non-adapted powdery mildews fail to complete their life cycle on *Arabidopsis*.

Genetic analyses identified components required for non-host resistance against powdery mildew. For example, forward genetic screens yielded four *Arabidopsis* mutants, (*pen1* through *pen4*) showing enhanced penetration rates, indicating that the corresponding wild type genes are essential for the non-host resistance phenotype (Collins et al., 2003; Lipka et al., 2005; Stein et al., 2006). *PEN1* encodes a syntaxin (SYP121) that mediates fusion of secretory vesicles with the plasma membrane, whereas the products of *PEN2*, a glycosyl hydrolase, and *PEN3*, an ATP-binding cassette (ABC) transporter, are predicted to load secretory vesicles with toxic compounds (Collins et al., 2003; Lipka et al., 2005; Stein et al., 2006; Micali et al., 2008). Thus, the cooperative action of PEN proteins contributes to pre-invasion/penetration resistance. In addition, post-invasion defense mechanisms restrict pathogen growth after haustorium formation. Genes encoding ENHANCED DISEASE SUSCEPTIBILITY 1 (EDS1), PHYTOALEXIN DEFICIENT 4 (PAD4), and SENESCENCE ASSOCIATED GENE 101 (SAG101) are essential defense components required for basal defense and execution of race-specific resistance mediated by a subset of *R* genes (Wiermer et al., 2005; Dodds and Rathjen, 2010). In the *eds1*, *pad4*, and *sag101* mutants the penetration rates of powdery mildews were not significantly different from the wild type, whereas in the double mutants *pen2 eds1* and *pen2 pad4* the non-adapted fungus was able to develop secondary hyphae while the HR occurred less frequently; in the triple mutant *pen2 pad4 sag101* non-host resistance was effectively abolished and the fungus could form microcolonies and complete its life cycle (Lipka et al., 2005; Stein et al., 2006). Thus, the removal of both defense layers, the PEN-mediated penetration resistance and the EDS1/PAD4-controlled post-invasion resistance makes *Arabidopsis* fully susceptible to non-adapted powdery mildews such as *E. pisi* (Lipka et al., 2005; Stein et al., 2006).

Biochemical and molecular analyses, complementing the genetic approaches, demonstrated that SA, JA, and ethylene signaling components could contribute to powdery mildew resistance (Reuber et al., 1998; Ellis et al., 2002; Zimmerli et al., 2004; Liu et al., 2005). In addition, the free radical NO has emerged as a signaling molecule in plant defense and its rapid production is strongly triggered after infection of plants with diverse pathogens (Delledonne et al., 1998; Leitner et al., 2009; Bellin et al., 2012). In fact, NO mediates signaling during numerous physiological processes and stress responses (Besson-Bard et al., 2008), but notably it participates, cooperatively with ROS, in the activation of HR cell death during incompatible plant-pathogen interactions (Delledonne et al., 1998; Zeier et al., 2004; Yoshioka et al., 2011). The formation of NO during plant defense frequently shows a biphasic temporal pattern, with a strong initial burst for a few minutes after infection or elicitor treatment, which is followed by a second sustained increase for several hours, and this latter increase seems to correlates with the disease resistance phenotype (Zeier et al., 2004; Mur et al., 2006). In tomato, infection with the powdery mildew fungus, *Oidium neolycopersici, *caused a rapid NO burst in both susceptible and resistant cultivars, but a sustained NO production was only observed in resistant tomato cultivars, which occurred simultaneously with a drastic increase in ROS, followed by HR cell death of penetrated epidermal cells and retardation of pathogen growth (Mlíčková, 2004; Piterková et al., 2009). Similarly, infection of barley with the powdery mildew fungus *Blumeria graminis *f. sp. *hordei* resulted in a transient NO burst in epidermal cells, which preceded HR cell death (Prats et al., 2005). However, how the NO and ROS signals are integrated and how precisely they mediate disease resistance remains unknown (Yoshioka et al., 2011).

Despite extensive research efforts, the precise function of NO in the plant immune response remains enigmatic. In particular, the route(s) of NO production in plants are still not unequivocally identified (Besson-Bard et al., 2008; Bellin et al., 2012). Mostly two enzymatic sources of NO are considered: (1) NO synthase (NOS; or NOS-like activity) catalyzing the NADPH-dependent oxidation of arginine as in animal cells, and (2) nitrate reductase (NR) catalyzing NO formation *via* nitrite (Yamasaki and Sakihama, 2000; Guo et al., 2003; Besson-Bard et al., 2008). In addition, NO may arise from other oxidative reactions (enzymatic and non-enzymatic) and it may be rapidly and easily converted to other reactive nitrogen species, because NO and ROS production often occur simultaneously (Besson-Bard et al., 2008; Bellin et al., 2012). Although mutant *Arabidopsis* plants with impaired NO production are more susceptible to pathogens (Zeidler et al., 2004; Modolo et al., 2005), it is still not clear whether NO is a signal, controlling downstream defense responses, or a disease symptom functioning as a proxy of active defense, or because of its reactive nature directly impairs pathogen growth and development. By taking advantage of the genetic resources available for the model plant *Arabidopsis thaliana*, we investigated the role of NO in the interaction with the adapted and non-adapted powdery mildew fungi, *G. orontii* and *E. pisi, *respectively. Our results show that NO has the capacity to function as signal molecule and to mediate other defense responses, but an additional direct impact on pathogen growth and development cannot be excluded.

## MATERIALS AND METHODS

### PLANT LINES AND GROWTH CONDITIONS

In this study we used the *Arabidopsis thaliana* Col-0 genotype, the single mutants *eds1-*2 (Bartsch et al., 2006), *pen2 *(Lipka et al., 2005), *nos1/noa1* subsequently referred to as *noa1* (Guo et al., 2003; Moreau et al., 2008), the double mutants *pen2 eds1-*2 (Lipka et al., 2005), *nia1 nia2* (Wilkinson and Crawford, 1993), and the *Arabidopsis* line *35S::nNOS* expressing rat neuronal NOS (nNOS) under the control of CaMV 35S promoter (Shi et al., 2012), all in the Col-0 genetic background. *Arabidopsis* seeds were surface-sterilized and placed on half-strength MS basal salt medium (Murashige and Skoog, 1962) containing 0.5% sucrose and 0.8% phytagel. After stratification for 2 days at 4°C in the dark, plates were vertically mounted under continuous yellow light for 3–4 days. Seedlings were transferred to pots with soil substrate and plants grown for 18 days at a day/night cycle of 10/14 h in a growth chamber at 22°C/20°C day/night temperature and a relative humidity of 60%.

### PLANT INOCULATION AND MICROSCOPIC ANALYSIS

Four week old plants were inoculated by brushing onto rosette leaves conidia of the *Golovinomyces orontii* isolate MPIPZ or conidia of *Erysiphe pisi* isolate MPIPZ, which where propagated as previously described (Lipka et al., 2005; Göllner et al., 2008; Weßling and Panstruga, 2012).). Inoculated plants were returned to the growth chamber for the indicated times. To visualize fungal structures, leaves were harvested, treated with ethanol:acetic acid 3:1 (v/v) to remove chlorophyll and stained with Coomassie Brilliant Blue as described previously (Göllner et al., 2008). Bright field images were taken with an AxioImager.A2 microscope equipped with an AxioCam HRc camera system (Carl Zeiss, Jena, Germany). All experiments were repeated twice and 5–10 images were analyzed per replicate and genotype using at least four different leaves each. A minimum of 100 fungal interaction sites was analyzed per leaf and the percentage of successful penetration events was calculated.

### SPORE COUNTS

The success of leaf colonization by powdery mildews was evaluated by counting spores on inoculated leaves as previously described (Weßling and Panstruga, 2012). At 7 day post-inoculation, four leaves were harvested per genotype, submerged in 5 ml water and spores were released by thoroughly vortexing. The solution was filtered through Miracloth (Merck, Darmstadt, Germany) to remove large debris and spores were counted in a Neubauer hemocytometer (Marienfeld, Lauda-Königshofen, Germany). Spore counts were normalized to the leaf fresh weight.

### DETERMINATION OF NO CONTENT

The intracellular NO level was determined by using the cell-permeable, fluorescent probe diaminofluorescein-FM diacetate (DAF-FM DA; Sigma-Aldrich, Taufkirchen, Germany), which after conversion by cytosolic esterases to DAF-FM can rapidly react with NO to form the corresponding green fluorescent triazole (DAF-FM T; Suzuki et al., 2002; Gould et al., 2003). Leaves were infiltrated with 10 mM Tris (pH 6.5) containing 10 μM DAF-FM DA (added from a 10 mM stock in DMSO) for 30 min in the dark, rinsed with water and mounted on microscopic slides. Specimen were examined with a confocal laser scanning microscope LSM 510 Meta (Zeiss, Oberkochen, Germany) equipped with an argon mixed gas laser and a filter set (excitation 488 nm, emission 515 nm) for detection of green DAF-FM T fluorescence. Serial confocal optical sections were taken at a step size of 1 μm and these Z-stacks, reconstructed into three-dimensional images, were used to quantify the NO-specific fluorescence at infection sites within areas defined by circles of approximately 50 μm in diameter by determining pixel densities with the open source software Image-J^1^. Parameters for confocal microscopy, in particular laser and detector settings, were identical for all experiments and appropriate control samples were always included. To verify that the recorded increase in fluorescence is dependent on NO accumulation, we pretreated leaves with NO scavenger (e.g., 200 μM cPTIO, see below) prior infection, which in all cases abolished DAF-FM-based fluorescence. Auto fluorescence at infection sites of control leaves was also recorded and subtracted from all experimental samples. For each data point a minimum of 20 infection sites from four different leaves taken from two different plants was analyzed and each experiment was repeated twice.

### TREATMENT WITH CHEMICALS

To conditionally modulate endogenous NO levels, leaves were treated with various chemicals known to release NO, such as 200 μM *S*-nitrosoglutathione (GSNO) or 100 μM *S*-nitroso-N-acetyl-D-penicillamine (SNAP), or compounds scavenging NO or impairing its formation, such as 200 μM 2-(4-carboxyphenyl)-4,4,5,5-tetramethylimidazoline-1-oxyl-3-oxide potassium salt (cPTIO), 100 μM L-N^ω^-nitro-arginine methyl ester (L-NAME) or 100 μM okadaic acid (OA). All compounds (obtained from Sigma-Aldrich, Taufkirchen, Germany) were dissolved in DMSO (10 μM) and the indicated, effective working solutions in 10 mM MgCl_2_ freshly prepared immediately before infiltration into leaves with a syringe. Plants were incubated with chemicals for 2 h before inoculation with the powdery mildews.

### QUANTIFICATION OF SALICYLIC ACID

Salicylic acid (SA) content in leaves was quantified as previously described (Straus et al., 2010). SA was extracted from 100 to 150 mg plant material in 1 ml chloroform/methanol/water (1:2:0.3) containing 160 pmol 2-hydroxybenzoic-3,4,5,6-d_4_ acid (SA-d_4_; Campro Scientific, Berlin, Germany) as internal standard. After shaking for 10 min at 70°C samples were centrifuged and re-extracted with 0.5 ml chloroform/methanol (1:2). After phase separation through the addition of 0.5 ml H_2_O the polar extract was dried. Samples were dissolved in 1 ml sodium acetate (pH 5) and divided equally for total and free SA analysis. For total SA, samples were treated with almond β-glucosidase (Sigma-Aldrich, Taufkirchen, Germany) for 3 h at 37°C. Both, total and free SA samples were acidified with 30 μl 10% trifluoroacetic acid (TFA) and extracted twice with 0.6 ml ethyl acetate/hexane (3:1). Following evaporation of organic solvents, analytes were derivatized wi-th 80 μl pyridine/N-methyl-N-(trimethylsilyl)trifluoroacetamide (1:1; Sigma-Aldrich, Taufkirchen, Germany) and 1 μl was injected into a gas chromatograph coupled to a mass spectrometer (GC-MS; Agilent Technologies)^2^. Masses of SA-d_4_ (*m*/*z* 271) and SA (*m*/*z* 267) were detected by selected ion monitoring and quantified using the Chemstation software from Agilent.

### QUANTITATIVE REAL-TIME PCR

Relative transcript levels of PR1 were determined by quantitative real-time PCR (qRT-PCR) according to established protocols (Schmittgen and Livak, 2008; Weßling and Panstruga, 2012). Total RNA was extracted from 100 mg leaf issue and reverse transcribed to generate first-strand cDNA with the Super-Script First-Strand Synthesis System for RT-PCR (Invitrogen, Darmstadt, Germany) using oligo(dT) and random hexamer primers according to the manufacturer’s protocol. All qPCR assays were performed with cDNA corresponding to 100 ng RNA using the iQ^TM^ SYBR^®^ Green Supermix Kit (Biorad)^3^ on the iQ5 Real-Time PCR Detection System (Bio-Rad Laboratories, München, Germany). We used gene-specific primers at a final concentration of 0.1 μM and expression of the actin gene (At3g18780) served as control (PR1-forward: TTCTTCCCTCGAAAGCTCAA, PR1-reverse: AAGGCCCACCAGAGTGTGTATG; actin-forward: CGGTAACATTGTGCTCAGTGGTGG; actin-reverse: CAACGACCTTAATCTTCATGCTGC). qPCR assays were carried out in three technical replicates per sample according to the following conditions: denaturation at 95°C for 2 min, 40 repeats at 95°C for 20 s, 56°C for 30 s, and 72°C for 25 s. Relative expression levels were calculated using the ΔΔC_T_ method (Schmittgen and Livak, 2008) and normalized to the expression in uninfected control plants (0 hpi).

## RESULTS

### NO ACCUMULATION IN ARABIDOPSIS LEAVES UPON INOCULATION WITH POWDERY MILDEWS

**FIGURE 1:**
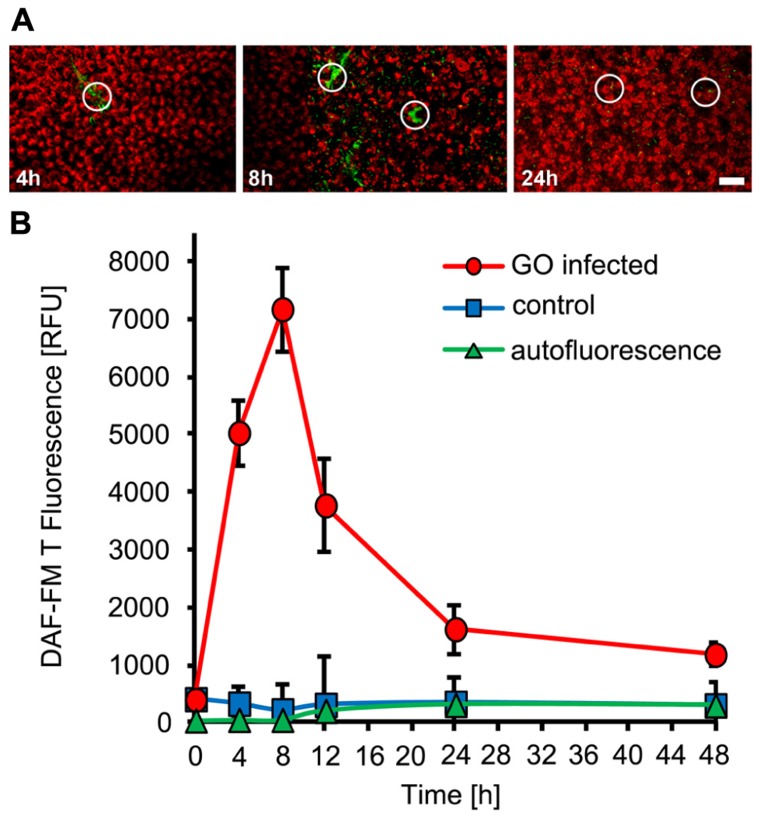
**NO accumulation in *Arabidopsis* leaves upon inoculation with the adapted powdery mildew fungus, *Golovinomyces orontii***. Leaves of *Arabidopsis thaliana* Col-0 were harvested at the indicated times after inoculation and used to detect intracellular NO by infiltration of the NO sensitive dye DAF-FM DA. **(A) **Time series of confocal images (taken at 4, 8, and 24 h post-inoculation) showing focused NO accumulation, as indicated by the green fluorescence, at the powdery mildew infection sites (white circles). The red color is due to chlorophyll fluorescence. **(B)** Time course of NO accumulation at fungal infection sites (red circles) and corresponding areas of non-infected control leaves (blue squares). NO was quantified by integrating the pixel intensity of green DAF-FM T fluorescence in three-dimensional optical reconstructions of infections sites (area defined by circles). Autofluorescence at infection sites was quantified without prior staining with DAF-FM DA (green triangles). Each data point reflects the mean (±SD) of 20 infection sites taken from four different leaves of two different plants. Bar = 50 μM.

**FIGURE 2:**
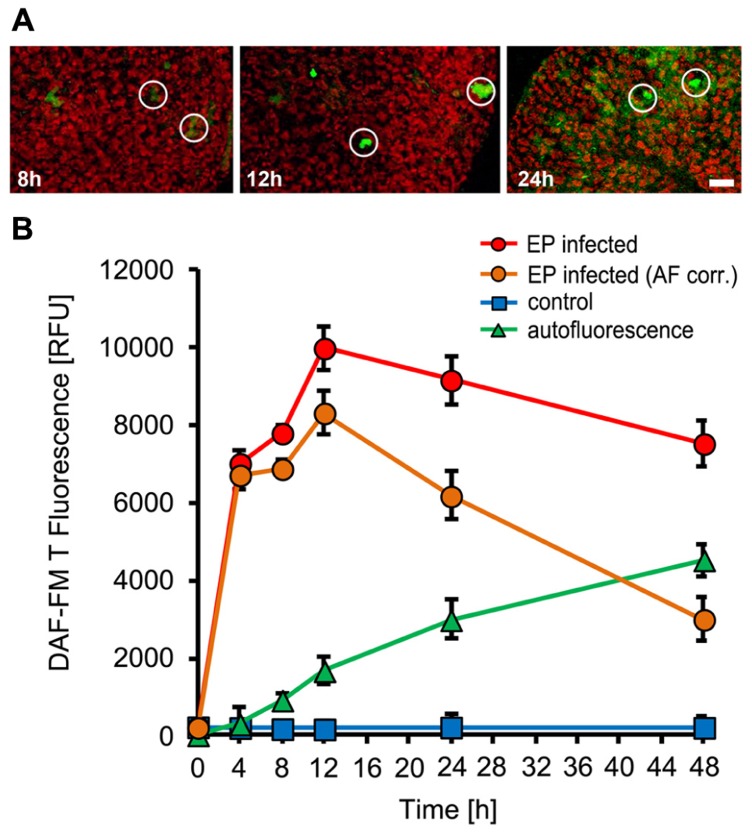
**NO accumulation in *Arabidopsis* leaves upon inoculation with the non-adapted powdery mildew fungus, *Erysiphe pisi***. Leaves of *Arabidopsis thaliana* Col-0 were harvested at the indicated times after inoculation and used to detect intracellular NO by infiltration of the NO sensitive dye DAF-FM DA. **(A)** Confocal images of powdery mildew infection sites (white circles) taken at 8, 12, and 24 h post-inoculation. **(B)** Time course of NO accumulation at infection sites (red circles) and in non-infected control leaves (blue squares). Autofluorescence at infection sites was quantified without prior DAF-FM DA staining (green triangles) and these values were used to correct NO levels (orange circles). All data represent the mean (±SD) of 20 infection sites taken from four different leaves of two different plants. Bar = 50 μM.

To monitor NO production during the interaction of *Arabidopsis thaliana* with powdery mildew fungi, we used the cell-permeable dye DAF-FM DA (4-amino-5-methylamino-2,7-difluorofluorescein-FM diacetate), which is an established, specific probe for the detection of intracellular NO (Suzuki et al., 2002; Gould et al., 2003), in combination with confocal laser-scanning microscopy. When loaded into plant cells, DAF-FM DA is converted by cytosolic esterase to DAF-FM, which can react with N_2_O_3_, originating from oxidation of NO, to form the green fluorescent DAF-FM triazole derivative. When *Arabidopsis *plants (Col-0) were inoculated with the adapted powdery mildew *G. orontii*, rapid and localized NO accumulation was demonstrated by confocal laser scanning microscopy, which is restricted to few directly affected cells (**Figure 1A**). Quantitative analysis revealed a strong increase in NO amounts at the infection sites reaching maximum levels at 8 h post-inoculation followed by a rapid decrease thereafter (**Figure 1B**). The peak of NO formation coincided in timing with appressoria formation by *G. orontii* primary hyphae on the leaf surface, which initiates breaching of epidermal cell walls and precedes the formation of infection hyphae. Plants inoculated with the non-adapted powdery mildew fungus, *E. pisi*, showed a similar spatial pattern of NO formation, again restricted to few cells around infection sites (**Figure 2A**). However, the time course was delayed (maximum at 12 hpi) and the overall amounts of NO accumulating at infection were slightly higher when compared to *G. orontii* infection (**Figure 2B**). The incompatible interaction of *Arabidopsis* with non-adapted *E. pisi *is characterized by the development of rapid HR cell death of infected cells, which is associated with strong autofluorescence and therefore may interfere with NO detection and systematically distort its quantification. We examined the autofluorescence in infected tissue without DAF-FM staining and observed a continuous increase over time, which was used to correct the determined NO levels accordingly (**Figure 2B**). Obviously, NO quantification is primarily distorted at late time points (**Figure 2B**). By contrast, only low values of autofluorescence were recorded following inoculation with *G. orontii* and hence, the NO quantification was not affected (**Figure 1B**). From these infection studies it is evident that NO accumulation is a rapid, localized defense response and the rapid decline of initially high values in the compatible interaction of *Arabidopsis* with *G. orontii* may suggest that the adapted powdery mildew has developed strategies to remove NO or suppress its excessive accumulation.

### NO FORMATION IN ARABIDOPSIS MUTANTS WITH IMPAIRED DISEASE RESISTANCE

**FIGURE 3:**
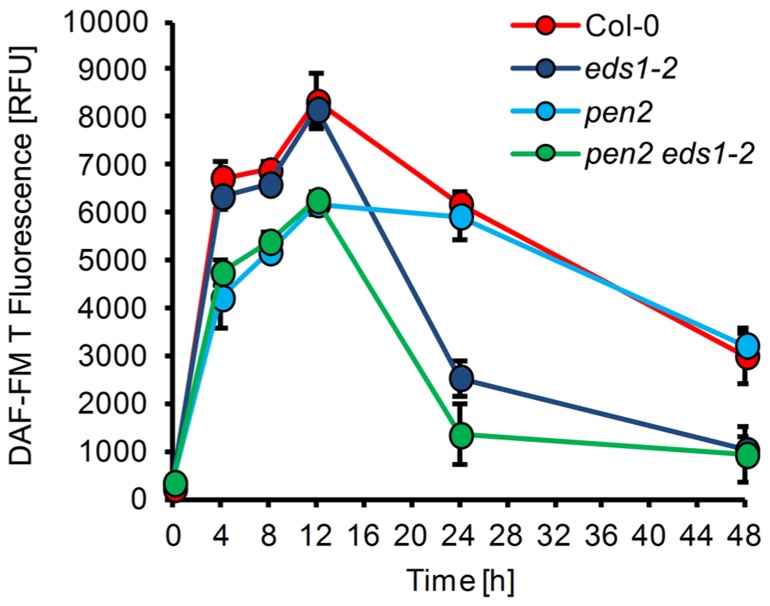
**NO formation in *Arabidopsis* mutants with impaired disease resistance.** Leaves of different *Arabidopsis thaliana* genotypes, Col-0 (red), *eds1-2* (purple), *pen2* (blue), *pen2 eds1-2* (green), were inoculated with the non-adapted powdery mildew *Erysiphe pisi* and harvested at the indicated times for quantification of NO formation by integration of DAF-FM T fluorescence at infection sites. (For experimental details, see Figure1). Values represent the mean (±SD) of 20 infection sites taken from four different leaves of two different plants.

To further explore the potential function of NO in plant immunity, we determined NO formation in *Arabidopsis* mutants that are impaired in their defense. First, we tested the *Arabidopsis* *pen2* mutant, which is compromised in penetration resistance toward non-adapted powdery mildews, such as *E. pisi*. In *pen2* NO formation essentially followed a similar time course as in wild type plants, with the exception that up to 12 h the absolute amounts are 25–30% lower (**Figure 3**). Since at 24 h post-inoculation the penetration frequency of *E. pisi* on *pen2* plants is drastically increased (60–80% of the interaction sites), this early reduction in NO correlates with and may be responsible for the complete loss of resistance and successful invasion of the mutant (**Figure 5A**; Lipka et al., 2005). The complete susceptibility of *Arabidopsis* toward adapted powdery mildew *G. orontii* is not further enhanced in the *pen2* mutant (not shown). 

Second, in the *eds1* mutant penetration resistance toward *E. pisi* is not impaired, but epiphytic hyphal growth, which occurs later during this interaction, is substantially increased, when scored at 7 days post-inoculation (Lipka et al., 2005). NO formation in *eds1* plants was unaffected during the early stages of interaction with *E. pisi* in comparison to wild type plants (**Figure 3**). However, drastically reduced NO levels were observed at 24–48 h post-inoculation, amounting to 40–50 % of wild type levels. Thus, suppression of NO formation or its removal at late infection stages may be causal for subsequent successful colonization of mutant tissue by the non-adapted powdery mildew fungus. The NO accumulation pattern in the *eds1 pen2* double mutant exactly matches the combined patterns of both single mutants, with reduced NO levels throughout the time period analyzed (**Figure 3**). Again, this correlates with impaired penetration resistance and even further enhanced epiphytic fungal growth on the leaf surface, resulting in microcolony formation as reported previously (Lipka et al., 2005).

### POWDERY MILDEW INFECTION OF ARABIDOPSIS MUTANTS WITH IMPAIRED NO PRODUCTION

**FIGURE 4:**
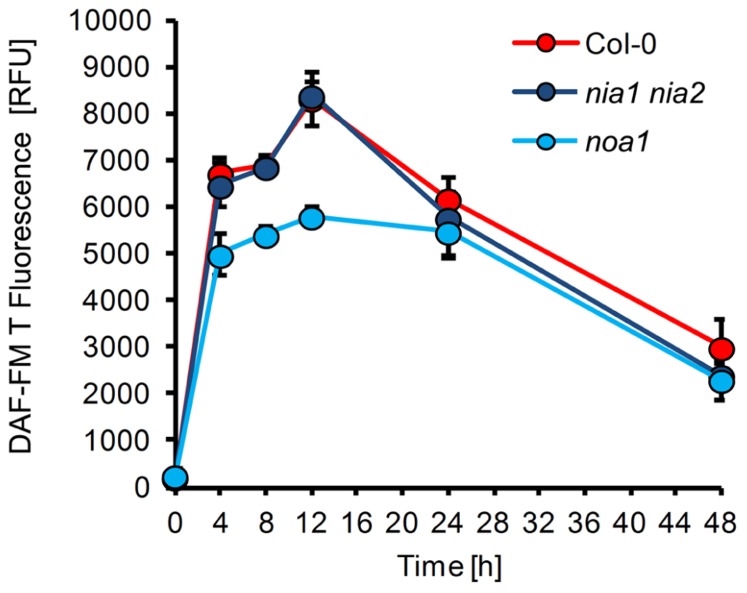
**NO formation in *Arabidopsis* mutants defective in putative NO synthesis pathways.** Leaves of different *Arabidopsis thaliana* genotypes, Col-0 (red), *nia1 nia2* (purple), *noa1* (blue), were inoculated with the non-adapted powdery mildew *Erysiphe pisi* and harvested at the indicated times for quantification of NO formation by integration of DAF-FM T fluorescence at infection sites. (For experimental details see legend to Figure1). Values represent the mean (±SD) of 20 infection sites taken from four different leaves of two different plants.

**FIGURE 5:**
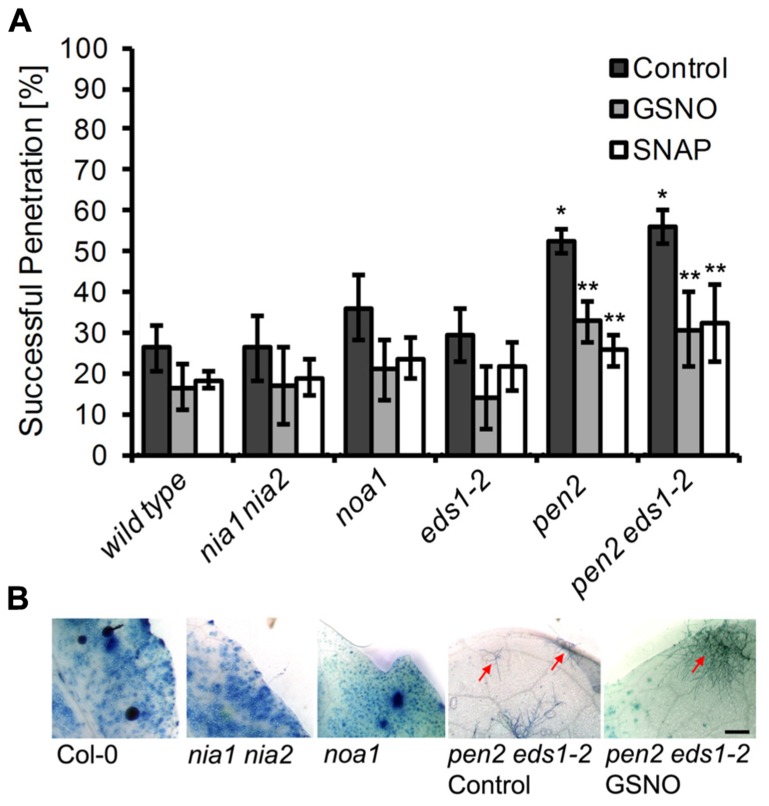
**Disease resistance phenotype of different *Arabidopsis* mutants to infection with *Erysiphe pisi*. (A)** Quantitative analysis of host cell entry (penetration rates), determined 48 h post-inoculation with *Erysiphe pisi *(black bars). The same analysis was carried out with leaves that were infiltrated with NO donors, 200 μM GSNO (gray bars) or 100 μM SNAP (white bars) 2 h prior to inoculation with *E. pisi *spores. Data represent the mean (±SD) of at least six leaves taken from two different plants. One asterisk indicates a significant difference (*p* < 0.01) between mutant and wild type plants, two asterisks indicates a significant difference (*p* < 0.01) between control and NO donor treatment. **(B) **Representative micrographs of infected leaves, harvested at 7 days post-inoculation, following staining with Coomassie Brilliant Blue to visualize fungal structures and host cells that have undergone HR cell death. The *Arabidopsis* mutant *noa1* and the double mutant *nia1 nia2* show no phenotypic difference to wild type plants (Col-0), whereas the double mutant *pen2 eds1-2* shows a lower frequency of HR cell death and sporadic microcolony formation (red arrow), which is not affected by pre-treatment with GSNO/SNAP. Bar = 1 mm.

In order to identify the metabolic route(s) of powdery mildew-induced NO formation, we used two *Arabidopsis* mutants with impaired capacity to synthesize NO. First, the contribution of NR was evaluated by analysis of the *nia1 nia2* double mutant, which is defective in both genes encoding active NR, NIA1, and NIA2 (Wilkinson and Crawford, 1993; Desikan et al., 2002). This mutant showed strongly reduced NO production upon infection with necrotrophic fungal pathogens and bacteria, such as *Botrytis cinerea, Sclerotinia sclerotiorum, *and* Pseudomonas syringae* (Modolo et al., 2006; Asai et al., 2010; Oliveira et al., 2010; Perchepied et al., 2010), but when inoculated with *E. pisi*, the pattern of NO formation in was indistinguishable from wild type plants (**Figure 4**). This result indicates that in the *Arabidopsis* *nia1 nia2* double mutant NO synthesis upon powdery mildew infection proceeds *via* an NR-independent pathway. Likewise, the resistance phenotype of the *nia1 nia2* double mutant was also not different from wild type plants, both allowing a rate of 26% successful penetration events (**Figure 5A**). This similarity in phenotypic appearance is also obvious from inspection of the tissue under the microscope (**Figure 5B**) and it is in accordance with the unaltered NO levels.

Second, we determined whether NO synthesis may originate from L-arginine *via* a NOS-like activity, which previously was believed to exist in plants and contribute to pathogen-induced NO formation (Guo and Crawford, 2005; Corpas et al., 2009; Asai et al., 2010). Although the *noa1* mutant is defective in a plastidic GTPase rather than NOS (Moreau et al., 2008; Gas et al., 2009), it shows reduced NO levels after bacterial infection or elicitor treatment (Delledonne et al., 1998; Zeidler et al., 2004). We found that upon inoculation with *E. pisi* the *noa1* mutant accumulated approximately 20–30% less NO in comparison to wild type plants (**Figure 4**). At the same time, the penetration rate of the powdery mildew fungus on the mutant increased slightly, but not significantly (from 26 to 36% compared to wild type), and also histological differences were not apparent (**Figures 5A and B**). The nearly unimpaired NO formation in the *nia1 nia2* double and *noa1* single mutant indicates that NO synthesis in *Arabidopsis* proceeds *via* a yet unknown route.

### IMPACT OF CHEMICALLY ALTERED OF NO LEVELS ON POWDERY MILDEW INFECTIONS

**FIGURE 6:**
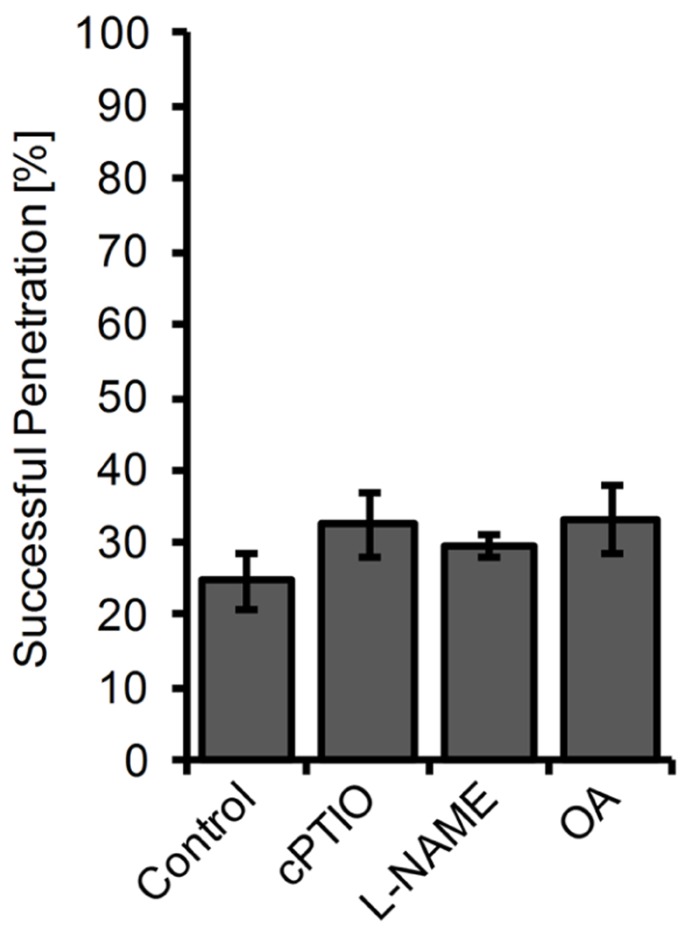
**Penetration rate of *Erysiphe pisi *on *Arabidopsis* is not affected by inhibitors of NO formation.** *Arabidopsis* wild type plants were treated for 2 h with 200 μM cPTIO, 100 μM L-NAME, 100 μM OA, or DMSO (control) in 10 mM MgCl_2_ before inoculation with the powdery mildew fungus *E. pisi* and host cell penetration was quantified 48 h post-inoculation. Data represent the mean (±SD) of all (at least 100) interactions sites analyzed on six leaves taken from two different plants.

Since the available mutants did not show significant alterations in cellular NO levels, we used a chemical approach to study the impact of NO accumulation on powdery mildew infections. Therefore, we first treated plants for 2 h with the NO scavenger cPTIO, the NOS inhibitor L-NAME, or the NR inhibitor OA, followed by inoculation with the non-adapted powdery mildew *E. pisi*. However, none of the tested compounds caused a significant increase in penetration frequency (**Figure 6**), suggesting that NO does not contribute to disease resistance and plant colonization by *E. pisi* is rather limited by other defense components. Conversely, plants treated with the NO donors GSNO or SNAP for 2 h prior to inoculation by *E. pisi* showed clearly reduced penetration rates, which is true for all plant genotypes tested, including wild type, *nia1 nia2*, and *noa1* (**Figure 5A**). In fact, both NO donors reduced the initial penetration phenotypes of the *pen2* and *pen2 eds1* mutant back to wild type levels, which could be explained by NO directly impairing fungal viability or indirectly enhancing other defense responses. However, the low frequencies of HR cell death and microcolony formation by *E. pisi* on the *pen2 eds1* double mutant was not affected by treatment with GSNO (**Figure 5B**).

**FIGURE 7:**
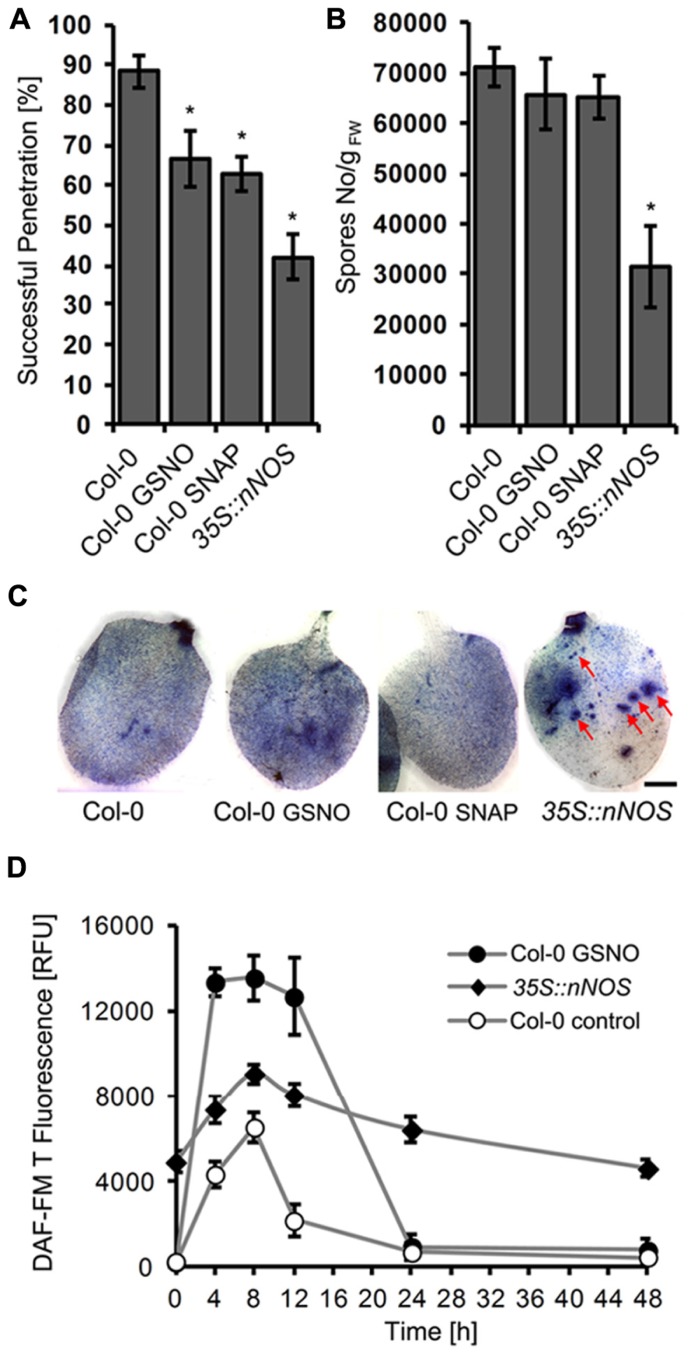
**Increased NO levels impair colonization of *Arabidopsis* by *Golovinomyces orontii.*** Six week-old *Arabidopsis* wild type (Col-0) was infiltrated with NO donors, 200 μM GSNO, 100 μM SNAP, or DMSO (control) 2 h before inoculation with *G. orontii *spores, whereas the *Arabidopsis* mutant *35S::nNOS* was inoculated without prior treatment. **(A)** Quantification of host cell entry rates determined 48 h post-inoculation (hpi). Data represent the mean (±SD) of at least six leaves taken from two different plants. **(B)** Production of spores at 7 days post-inoculation (dpi) normalized to leaf fresh weight. Data represent the mean (±SD) of at least four different leaves. Asterisks in both graphs (**A** and **B**) indicate a significant difference (*p* < 0.01) of treated plants/mutant relative to untreated wild type. **(C)** Representative micrographs of infected leaves, harvested at 7 days post-inoculation (dpi), following staining with Coomassie Brilliant Blue to visualize fungal structures and host cells that have undergone HR cell death. The *Arabidopsis* wild type is covered with sporulating colonies and this phenotype is not altered by prior treatment with GSNO or SNAP. By contrast, the *35S::nNOS line *is only partially colonized and developed intensely stained lesions (HR cell death) at high frequency (red arrows). Bar = 25 mm. **(D)** Time course of NO formation in *Arabidopsis* wild type (Col-0) and *35S::nNOS* plants upon inoculation with the adapted powdery mildew fungus, *G. orontii*. NO quantification at infection sites was carried out as described in Figure1. Values represent the mean (±SD) of at least 20 infection sites taken from four different leaves.

We also tested the impact of NO donors on infection of *Arabidopsis* by the adapted powdery mildew *G. orontii*. Treatment of leaves with GSNO or SNAP caused a significant reduction in penetration frequency (from 88 to 67 and 53%, respectively) as determined 2 days after inoculation (**Figure 7A**). However, this treatment did not affect the final outcome of this compatible interaction; when spores of the colonized leaves were counted 7 days after inoculation, we observed no significant differences in comparison to the control (**Figure 7B**). Both treated and untreated leaves were fully covered with sporulating *G. orontii* colonies (**Figure 7C**). One possible explanation for this result could be that NO donor treatment enhanced NO levels only for a short time period. Indeed, NO quantification revealed that GSNO-treated plants contained about two-fold higher NO levels at 8 h after infection with *G. orontii* in comparison to untreated control plants (**Figure 7D**). However, this increase rapidly vanished and at 24 h after inoculation, NO amounts declined to background level in both cases (**Figure 7D**).

Next we tested whether disease resistance is affected by sustained NO production. Therefore, we inoculated transgenic *Arabidopsis* plants expressing rat nNOS under the control of the CaMV 35S promoter (Shi et al., 2012). NO quantification confirmed that these plants contained drastically enhanced NO levels (compared to wild type), which transiently increased further after inoculation with *G. orontii* (**Figure 7D**). The phenotypic analysis revealed that these *35S::nNOS* plants showed a strongly reduced penetration rate after inoculation with *G. orontii* (42 vs 88% in wild type; **Figure 7A**), and also spore formation was significantly reduced (**Figure 7B**). Furthermore, the leaves of *35S::nNOS* plants were only partially colonization and unlike wild type plants developed necrotic lesions (**Figure 7C**). From these results we conclude that sustained NO formation has a positive impact on disease resistance, whereas temporal variation of NO concentrations is apparently insufficient.

### IMPACT OF ENHANCED NO LEVELS ON OTHER DEFENSE RESPONSES

**FIGURE 8:**
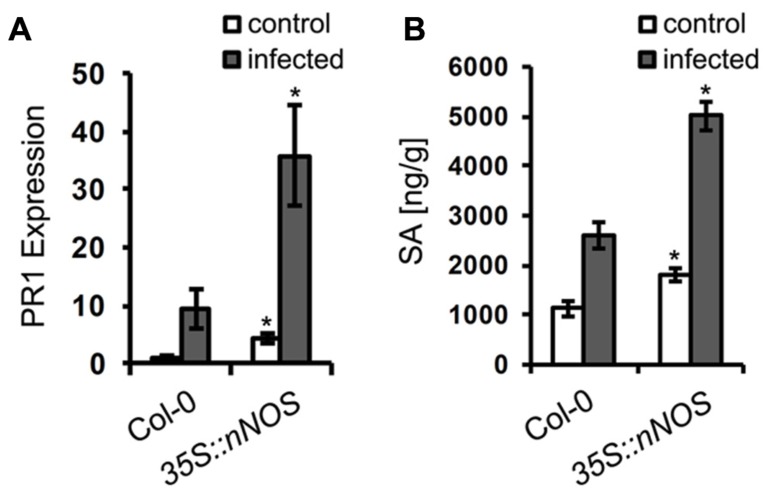
**Salicylic acid content and *PR1* gene expression are upregulated in *35S::nNOS* plants.** 6-week-old *Arabidopsis* plants, wild type and *35S::nNOS*, were inoculated with *Golovinomyces orontii* and leaves harvested for analysis at 24 h post-inoculation. **(A)** Relative expression of the *PR1* gene was determined by qRT-PCR (normalized to *actin* gene expression) and the value of unchallenged Col-0 plants set to 1. **(B)** Quantification of total SA levels. All values are the mean (±SD) of six plants taken from two separate experiments. In both graphs (**A** and **B**), asterisks indicate a significant difference (*p* < 0.01) of mutant relative to wild type plants.

Having shown a resistance phenotype of NO overproducing plants, we wanted to analyze whether this NO function is directly affecting the pathogen or whether it is mediated *via* other defense responses. We therefore analyzed two typical defense marker, expression of the *PR1* gene and accumulation of SA (Glazebrook, 2005; Vlot et al., 2009). In unchallenged *35S::nNOS* plants, PR1 gene expression was about 4-fold enhanced when compared to wild type plants (**Figure 8A**), and upon *G. orontii* infection it was about 10-fold induced in both genotypes (**Figure 8A**). Quantification of SA uncovered a similar pattern. Unchallenged *35S::nNOS* plants contained about 2-fold higher concentrations of total SA, which increased about 2.5-fold upon inoculation with *G. orontii*, as in wild type plants (**Figure 8B**). From these results we conclude that NO has the capacity to function as signal molecule to mediate other defense responses; however, a direct impact on pathogen growth and development cannot be dismissed.

## DISCUSSION

Initially, NO has been identified as regulator of numerous physiological responses in mammals and many years later similar biological functions of this molecule were uncovered in plants by demonstrating that it is an crucial component of the plant immune response (Delledonne et al., 1998; Durner et al., 1998). Importantly, NO participates, in cooperation with H_2_O_2_ (and other ROS), in activation of HR cell death in incompatible plant–pathogen interactions (Mur et al., 2006; Yoshioka et al., 2011; Bellin et al., 2012). This NO function has mainly been demonstrated when plants were infection with pathogenic bacteria, e.g., *Pseudomonas syringae* (Delledonne et al., 1998; Zeidler et al., 2004; Zeier et al., 2004; Modolo et al., 2005, 2006; Zago et al., 2006; Oliveira et al., 2010), but a contribution of rapid NO bursts to enhanced disease resistance has also been observed in various plants under attack by necrotrophic fungal pathogens, such as *Botrytis cinerea* or *Sclerotinia sclerotiorum* (Mur et al., 2006; Floryszak-Wieczorek et al., 2007; Asai and Yoshioka, 2009; Perchepied et al., 2010). By contrast, only few studies, focusing on the crop plants barley and tomato, have assessed the role of NO against biotrophic fungi such as powdery mildews (Prats et al., 2005; Piterková et al., 2009).

We wanted to elucidate the role of NO in the plant immune response toward biotrophic fungi by using the model plant *Arabidopsis thaliana* infected by the host-adapted powdery mildew *G. orontii* (compatible interaction) or the non-adapted powdery mildew *E. pisi* (incompatible interaction). We monitored NO formation with fluorescent dye DAF-FM DA, which not only allows quantification but can also provide insight into spatial accumulation patterns with cellular resolution. The specificity of DAF-FM DA for detection of NO has previously been demonstrated (Suzuki et al., 2002; Besson-Bard et al., 2008), although some caution is required to work under strictly aerobic conditions because NO reacts with the dye only in the presence of oxygen *via* the intermediate N_2_O_3_ (Arita et al., 2007). Applying this methodology, we could clearly show that *Arabidopsis*, similar to barley and tomato (Prats et al., 2005; Piterková et al., 2009), responds to powdery mildew infection with a rapid and transient NO accumulation, which is restricted to infection sites (**Figures 1** and 2). While the rapid accumulation of NO was similar in both, the compatible and the incompatible interaction, differences in the duration of elevated NO levels were apparent. In leaves infected with *G. orontii*, the NO level rapidly declined after the initial burst, which could be a consequence of active defense suppression mediated by effector molecules deployed by the host-adapted powdery mildew (O’Connell and Panstruga, 2006). By contrast, NO levels remained high for an extended time period following inoculation with *E. pisi* (**Figure 2**). Interestingly, the peak of NO accumulation (at 8–12 hpi) coincided with the time reported for formation of appressoria, which is a prerequisite for breaching the plant cell and presumably this process is also tightly linked with recognition of the pathogen by the host and coordinate defense activation.

The advantage of *Arabidopsis* as experimental system is the vast availability of various biological resources, experimental tools and acquired common knowledge. Therefore, we could access different mutants with defective pathogen defense. The analysis of two *Arabidopsis* mutants, *pen2* and *eds1*, which are impaired in pre-invasion and post-invasion defense mechanisms, respectively, are also differentially affected in their capacity of NO formation. In *pen2*, the temporal pattern of NO accumulation after inoculation with *E. pisi* was not affected, but the total amount was significantly reduced, which correlates with enhanced penetration rates of the fungus (**Figures 3** and 5), whereas extended hyphal growth and sporulation of the fungus was not supported (Lipka et al., 2005). In *eds1*, by contrast, the initial increase of NO was not affected, but a significant decrease occurred subsequently at late infection stages, which correlates with enhanced epiphytic fungal growth and formation of microcolonies (**Figures 3** and 5), and this phenotype is further enhanced in the *pen2 eds1* double mutant (Lipka et al., 2005). Although our results may suggest that impaired resistance is the result of reduced NO accumulation, we cannot necessarily infer such causal relationship. The gene products encoded by *PEN2* (glycosyl hydrolase/myrosinase) and *EDS1* (central regulator of plant defense) are functionally well characterized and not related to nitrogen or NO metabolism (Lipka et al., 2005; Wiermer et al., 2005; Bednarek et al., 2009). In fact, the inverse relationship cannot be excluded. Reduced NO levels in *pen2* and *eds1* may be the consequence of enhanced host colonization if the pathogen, *E. pisi*, has the capacity to suppress NO formation or to decompose the molecule.

The second type of mutant we used in our studies is affected in NO biosynthesis. In fact, two enzymatic pathways for NO synthesis have been described in plants (Besson-Bard et al., 2008). The first pathway includes a cytosolic NR, which produces NO *via* nitrite, but only with low efficiency (Yamasaki et al., 1999; Yamasaki and Sakihama, 2000). The *Arabidopsis* genome contains two NR genes, *NIA1* and *NIA2*, and their participation in NO formation is supported by the abolition of NR activity and NO production in the *nia1 nia2* double mutant (Desikan et al., 2002). The mutant is also defective in nitrogen assimilation, it contains decreased levels of nitrite and amino acids, and the impaired NO formation after pathogen infection can be rescued be application of nitrite (Modolo et al., 2005, 2006). The second pathway implicates a putative NOS-like enzyme catalyzing arginine-dependent NO formation in plants, although a homolog of animal NOS has not been identified in any sequenced plant genome (Corpas et al., 2006, 2009; Besson-Bard et al., 2008; Asai and Yoshioka, 2009). However, inhibitors of animal NOS also suppress NO formation in plants (Delledonne et al., 1998) and the *Arabidopsis noa1* mutant shows reduced NO levels (Guo et al., 2003; Guo and Crawford, 2005). Previously, this mutant was considered to be impaired in NOS, but recently it was demonstrated that the defective gene encodes a functional GTPase and the reduced NO levels are an indirect consequence of the mutation, impairing chloroplast functions, and therefore NOS was renamed to NO-ASSOCIATED PROTEIN 1 (NOA1) (Moreau et al., 2008; Gas et al., 2009). In any case, the *Arabidopsis *mutant *noa1* is not only impaired in NO production but is also more susceptible to infection by diverse pathogens, including *Pseudomonas syringae, Colletotrichum orbiculare *and* Sclerotinia sclerotiorum* (Zeidler et al., 2004; Asai et al., 2008; Perchepied et al., 2010). By contrast, our results clearly show that in the *nia1 nia 2* double mutant the time course and amount of NO accumulation after inoculation with *E. pisi* is not different from the wild type and in the *noa1* mutant the amount is only slightly reduced, to about 70–80% of the wild type level (**Figure 4**). More importantly, we found no significant difference in disease resistance of both types of mutant toward *E. pisi* in comparison to wild type. These results indicate that none of the two outlined pathways (NR or NOS) seems to contribute to NO formation in *Arabidopsis* following infection by the biotrophic fungus *E. pisi*. Instead, NO may originate from a yet unknown pathway(s) or from non-enzymatic reactions (Besson-Bard et al., 2008). Furthermore, a contribution of NO to disease resistance can neither be inferred nor excluded from this mutant comparison, because the NO levels were only insufficiently altered.

The lack of additional NO-deficient mutants required alternative strategies to unveil the origin of NO and to modulate its cellular amounts. We have chosen a chemical approach to alter the plant endogenous NO-levels. Pretreatment of leaves with L-NAME, a widely used animal NOS inhibitor, which also suppresses NO synthesis in plants (Barroso et al., 1999; Rasul et al., 2012), or the NR inhibitor OA (Rockel et al., 2002), did not significantly affect plant resistance toward *E. pisi*, and likewise the NO scavenger cPTIO had also no effect (**Figure 6**). This is in accordance with the infection phenotypes of the NO-deficient mutants, *nia1 nia2* and *noa1*, collectively suggesting that NO is not involved in mediating immune responses to biotrophic pathogens. However, the opposite approach, increasing endogenous NO level by treatment of leaves with NO donors GSNO or SNAP prior to infection with *E. pisi* resulted in enhanced penetration resistance (**Figure 5**). This response was observed in all *Arabidopsis* genotypes infected with the non-adapted powdery mildew *E. pisi*, and the penetration rates of the adapted powdery mildew *G. orontii* were also significantly impaired by NO donor treatment (**Figure 7A**). However, this increased penetration resistance, manifested at 2 days post-inoculation, did not translate into post-invasion resistance and, despite pretreatment, *G. orontii* was able to complete its life cycle and colonize the host, as evident at 7days post-inoculation (**Figures 7B and C**). The quantification of NO in these NO donor-treated plants revealed a transiently enhanced accumulation upon infection, which could explain the reduced penetration frequency. For *Colletotrichum coccodes* it is documented that NO delays *in vitro *germination of conidia (Wang and Higgins, 2005) and for the tomato powdery mildew *Oidium neolycopersici* it has been shown that the transition from conidia to hyphae is sensitive to NO (Piterková et al., 2011). However, our results also indicate that after the initial burst, when NO had declined to background level, *G. orontii* could obviously resume growth and eventually colonized the whole leaf (**Figure 7C**). Thus, *G. orontii* can apparently cope with NO and even an active role in modulating its amount by degradation/decomposition or synthesis cannot be dismissed. Indeed, several fungi have been shown to produce NO *in vitro* and *in vivo*, including *Pythium* sp., *Botrytic* sp., *Fusarium* sp., *Blumeria graminis* and *Magnaporthe oryzae*, but the functional significance is unknown and possible routes of synthesis unresolved (Conrath et al., 2004; Prats et al., 2008; Samalova et al., 2013).

Although NO can also stimulate fungal development and/or drive the infection process, our results, and most of the published data, rather support an adverse effect of NO on fungal growth. This is most evident from the analysis of the *35S::nNOS* expressing *Arabidopsis* line, which contained constitutively enhanced NO levels, in contrast to the transient burst that was achieved upon treatment with NO donors, and which rendered the plant more resistant to infection by *G. orontii *(**Figure 7**). This transgenic line was previously shown to also display enhanced resistance to *P. syringae* and various abiotic stresses (Shi et al., 2012). Since this enhance resistance is associated with accumulation of SA and enhanced expression of defense marker genes such as PR1 (**Figure 8**), and many others (Shi et al., 2012), it can speculated that fungal growth restriction is the result of multiple plant defense components that are mediated by NO. However, a direct impact of NO on fungal growth and development is not excluded.

The production of NO is a conspicuous feature of the plant immune response and many details of its synthesis are still hidden in the haze. By contrast, concerning the function of NO a picture is emerging that involves *S*-nitrosylation of cysteine thiols as pivotal regulatory mechanism for the activation of plant defense responses (Besson-Bard et al., 2008; Leitner et al., 2009; Bellin et al., 2012). Among the numerous proteins that are *S*-nitrosylated several important regulators of plant defense were identified, including the transcriptional co-regulator NON-EXPRESSOR OF PR1 (NPR1) mediating SA-dependent defense activation (Tada et al., 2008; Lindermayr et al., 2010), the SA-BINDING PROTEIN 3 (SABP3) involved in SA signaling and expression of resistance against pathogen infection (Wang et al., 2009), and the NADPH oxidase RBOHD mediating HR cell death development by synthesis of ROS (Yun et al., 2011). The activity of all these proteins was affected by *S*-nitrosylation, and although this modification mechanism involves additional components such as glutathione (GSH), GSNO, ROS, and other redox mediators, it is obvious that NO has the potential to play a crucial role in defense signaling. Therefore, it is not surprising that constitutively enhanced NO production leads to defense activation, e.g., SA accumulation and PR1 gene expression, and eventually results in elevated disease resistance, as we demonstrated for the *Arabidopsis 35S::nNOS* line (**Figure 8**) and others previous reported (Shi et al., 2012).

In conclusion, NO plays a pivotal role in the immune response of plants to attack by diverse microbial pathogens, not only bacteria and necrotrophic fungi (as previously reported), but also biotrophic powdery mildews (as our results show). We demonstrated that a key feature of pathogen-induced NO formation is the rapid and transient accumulation and by extending the time period of elevated NO levels by chemical or genetic manipulation, enhanced disease resistance could be achieved. However, the molecular mechanism of this NO bioactivity is still largely unknown and this is also true for the route(s) of NO synthesis during plant–microbe interactions. Our analysis of two NO-deficient *Arabidopsis* mutants (*nia1 nia2* and *noa1*) excluded NO production *via* the known NR and/or NOS-like pathways. Clearly, there is a need for additional genetic resources to unravel NO biosynthesis and function and therefore we initiated a genetic screen in search for new and/or alternative components that should help to uncover the origin and potential targets of this important signaling component.

## Conflict of Interest Statement

The authors declare that the research was conducted in the absence of any commercial or financial relationships that could be construed as a potential conflict of interest.

## Footnotes

1 http://rsb.info.nih.gov/ij/
